# A Prospective Study on Time to Recovery in 254 Injured Novice Runners

**DOI:** 10.1371/journal.pone.0099877

**Published:** 2014-06-12

**Authors:** Rasmus Oestergaard Nielsen, Lotte Rønnow, Sten Rasmussen, Martin Lind

**Affiliations:** 1 Department of Public Health, Section of Sports Science, Aarhus University, Aarhus, Denmark; 2 Orthopaedic Surgery Research Unit. Science and Innovation Center, Aalborg University Hospital, Aalborg, Denmark; 3 Department of Orthopaedics, Aarhus University Hospital, Aarhus, Denmark; University of Northampton, United Kingdom

## Abstract

**Objectives:**

Describe the diagnoses and the time to recovery of running-related injuries in novice runners.

**Design:**

Prospective cohort study on injured runners.

**Method:**

This paper is a secondary data analysis of a 933-person cohort study (DANO-RUN) aimed at characterizing risk factors for injury in novice runners. Among those sustaining running-related injuries, the types of injuries and time to recovery is described in the present paper. All injured runners were diagnosed after a thorough clinical examination and then followed prospectively during their recovery. If they recovered completely from injury, time to recovery of each injury was registered.

**Results:**

A total of 254 runners were injured. The proportion of runners diagnosed with medial tibial stress syndrome was 15%, 10% for patellofemoral pain, 9% for medial meniscal injury, 7% for Achilles tendinopathy and 5% for plantar fasciitis. Among the 220 runners (87%) recovering from their injury, the median time to recovery was 71 days (minimum  = 9 days, maximum  = 617 days).

**Conclusions:**

Medial tibial stress syndrome was the most common injury followed by patellofemoral pain, medial meniscal injury and Achilles tendinopathy. Half of the injured runners were unable to run 2×500 meters without pain after 10 weeks. Almost 5% of the injured runners received surgical treatment.

## Introduction

Running-related injuries are common in novice-, recreational- and elite runners. According to a recent systematic review by Lopes et al [1], medial tibial stress syndrome, Achilles tendinopathy and plantar fasciitis were the most common diagnoses in recreational and elite runners, while Taunton et al [2] found patellofemoral pain to be the most frequent diagnose among 2002 runners attending an examination in the primary care.

In novice runners, however, limited insight is available in the literature into the type of running-related injuries. Buist et al [3] reported the lower leg and the knee to be the anatomical sites most often affected by running-related injuries in novice runners. An observation supported by Taunton et al [4]. Despite this valuable information, no data was collected on the types of injuries diagnosed by a health professional. Additionally, there is a lack of knowledge on the time to recovery from injuries sustained by novice runners. No studies have, to our knowledge, investigated the time to recovery in novice runners using a prospective setup.

In a recent prospective cohort study entitled the Danish Novice Running project (DANO-RUN) [5], 933 healthy novice runners were followed and diagnosed by a health professional in case of injury. Injured participants were then followed prospectively during their recovery. This dataset enabled us to bridge the gap on the types of running-related injuries sustained by novice runners and enables us to describe the time to recovery of such injuries.

## Methods

The DANO-RUN study was a 1-year prospective follow-up study aiming at characterizing risk factors for injury in novice runners. Present paper describes a sub-analysis of the injured novice runners included in the DANO-RUN study. The procedure for enrolment, inclusion and exclusion criteria and main purpose of the DANO-RUN study has been presented elsewhere[5]–[7]. All participants provided informed written consent prior to inclusion and the research was conducted in accordance with the Helsinki Declaration. The Scientific committee, Central Denmark Region evaluated the protocol (M-20110114) but waived the request for approval because observational studies, according to the Danish law, do not require an ethical approval. The Danish Data Protection Agency approved the study.

A novice runner was defined as a person who had not been running on a regular basis for the past year. The cut-off to define a regular basis was set at 10 km of the total running distance in all training sessions during the past year prior to inclusion. If a total of 10 km was exceeded a person was ineligible for inclusion. For instance, a person was included if he/she had been running a total of 3 times 2 kilometers in the past year and excluded if he/she had been running 5 times 4 kilometers. A running-related injury was defined as any musculoskeletal complaint of the lower extremity or back caused by running, which restricted the amount of running (distance, duration, pace, or frequency) for at least 1 week. This definition of injury was a modified version of the injury definition used in the studies on novice runners [3], [8], [9]. The date of injury occurrence was based on the anamnesis where the injured runner was asked to recall the date at which the symptoms started.

Prior to the study, no consensus-based definition of time to recovery was found. The authors defined recovery from injury as no pain in the affected anatomical location following two consecutive running sessions of at least 500 meters. The time to recovery was calculated as time in days from injury occurrence to complete recovery. In the case a participant was free of pain in activities of daily living but refused to run in order to evaluate on their symptoms, the recovery was defined as the day they were free of pain.

All injured participants attended a clinical examination in case of injury. At the examination, the participant was examined and diagnosed, preferably no later than 1 week after the participant had requested an examination. In most examinations (more than 80%), at least two physiotherapists (of four assisting in the study) diagnosed the injured participant based on a consensus agreement. A standardized examination procedure was used in each of the following anatomical locations: foot/ankle, lower leg, knee, thigh, hip and back. Furthermore, guidelines for diagnostic criteria were used to classify the injuries into specific diagnoses/types of injuries. These non-validated guidelines were developed by the DANO-RUN research group prior to the study (Material S1). At each examination, the injured runner was asked if they believed the injury was caused by running. If they said “yes” the injury was included in the analysis, while the injury was excluded if they said “no”. If they said “no” the injury was not registered in the database and it is, therefore, not possible to present details about these types of injuries.

In case the physiotherapist was unable to diagnose the injured runner at the clinical examination or the participant did not recover as expected after being diagnosed, an additional examination including diagnostic imaging (most often MRI) was performed. Such examination was provided in approximately 25% of all injuries and was always offered if the physiotherapist at the first examination diagnosed injuries like medial meniscal injury, osteoarthritis or stress fractures. The additional examination and diagnostic imaging were performed at the Division of Sports Traumatology at Aarhus University Hospital, Denmark. Based on the clinical examination(s), the diagnoses were registered for all injuries occurring from inclusion in the study and in the following 1-year period. In cases where the examiners (Physiotherapist and medical doctors) were in doubt and the diagnostic imaging was negative, the injury was classified as unknown.

After the clinical examination, all injured participants were followed prospectively and contacted by phone or mail once every 2 to 3 weeks to follow-up on injury status. In case the participants recovered from injury, questions regarding the use of medication (yes/no), treatment assistance from health professional (yes/no), surgical treatment (yes/no) and missed days from work (number) were asked. In addition, participants had to report their motivation to start running again after having had an injury and this information was then dichotomized into: 1) Less motivated or not at all motivated or 2) motivated or very motivated. If a participant completely recovered from injury and sustained an additional running-related injury afterwards, the participant had to attend a clinical examination again. The possibility of clinical examinations was stopped after the participants had been included in the DANO-RUN study for 1-year. The follow-up on injured participants were stopped February 2013, eighteen months after the first participants were enrolled at a baseline investigation and 7 months after the last participants completed follow-up.

Descriptive data were presented as counts and percentage dichotomous or categorical data. Data on time to recovery were presented as medians and the range between minimum and maximum because data on injury were considered non-parametric evaluated by histograms and quartile-quartile plots. Contra wise, data on the log scale was normally distributed. Therefore, the student's *t*-test was used to test if time to recovery (days) was different between those motivated to take up running again after having had an injury compared with those being less motivated. The Wilcoxon rank sum test was used to test if the time to recovery was different across gender and across age (dichotomized into less than or above 40 years because masters runners typically are defined as persons above 40 and face subtle differences in injury rate and location[10]) because the data were not normally distributed on original scale or on the log scale. All analyses were performed using STATA/SE version 12.1. A result was considered significant at p<0.05.

## Results

A total of 254 of the 933 novice runners included in the DANO-RUN study sustained at least one running-related injury during follow-up. Of these, 25% of the injuries occurred within the first 37 kilometers of running, 50% within the first 119 kilometers and 75% within the first 201 kilometers. Additionally, the median number of running sessions to injury occurrence was 36 with an inter-quartile range from 15 to 56. Three of the injured participants ran an average distance above 16 kilometers over the 1-year follow-up (kilometer prior to injury and after recovery all together) and 18 had an average distance above 10 kilometers.

**Figure 1:**
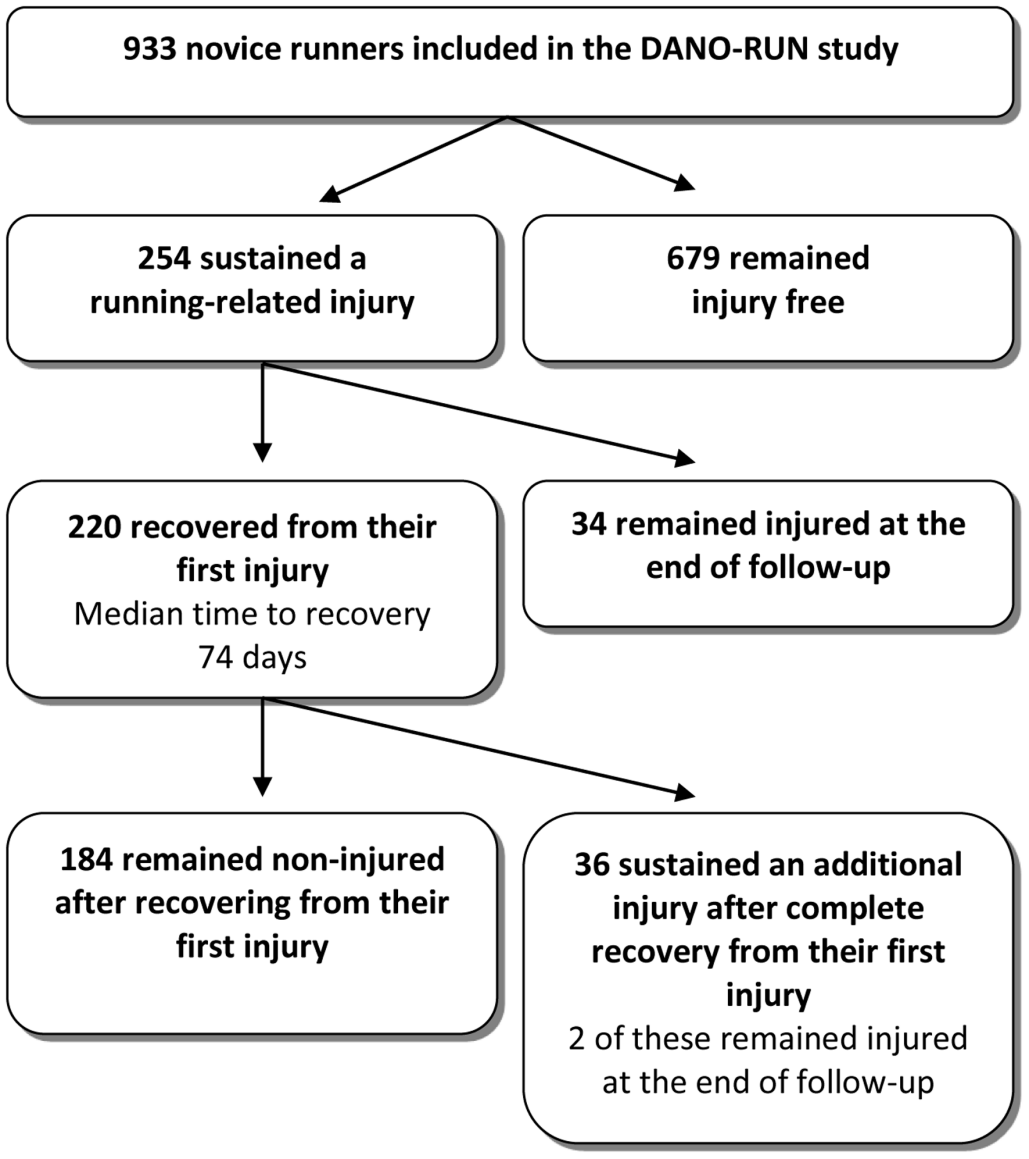
Flow-chart of the 254 injured participants.

The proportions of runners sustaining different types of injuries are presented in Table 1. A total of 220 (86.6%) participants completely recovered from their running-related injury. A majority of these took up running again, but 36 (16.4%) sustained an additional running-related injury of which 13 (36%) were diagnosed with the same injury as they had the first time. A flow-chart is presented in Figure 1.

**Table 1:** Descriptive data on the types of running-related injuries.

Injury type	All injuries (n = 254)				
	Count	Gender (Males)	Age (Above 40)	Bilateral injuries	Non-recovered injuries
	n (%)	n (p)	n (p)	n	n (%)
Medial Tibial Stress Syndrome	38 (15)	18 (0.87)	8 (<0.01)*	29	1 (2.6)
Patellofemoral Pain	26 (10)	11 (0.56)	8 (0.08)	7	4 (15)
Meniscal injury (Medial)	23 (9)	10 (0.68)	7 (0.09)	0	6 (26)
Achilles tendinopathy	18 (7)	9 (1.00)	4 (0.03)*	4	2 (11)
Plantar fasciitis	12 (5)	6 (1.00)	6 (1.00)	1	3 (25)
Soleus injury	12 (5)	6 (1.00)	5 (0.77)	1	0 (0)
Ilio-Tibial Band Syndrome	11 (4)	1 (0.01)*	2 (0.07)	1	0 (0)
Patella tendinopathy	11 (4)	3 (0.23)	7 (0.55)	1	1 (9)
Gastrocnemius injury	9 (3)	3	2	0	0 (0)
Gluteus Medius injury	8 (3)	3	5	0	3 (37.5)
Hamstring	8 (3)	4	2	0	2 (25)
Ankle inversion	7 (3)	3	2	0	2 (29)
Compartment (lower leg)	6 (2)	2	3	4	1 (17)
Iliopsoas injury	5 (2)	1	2	2	0 (0)
Hip trochanteric bursitis	4 (1)	1	0	1	1 (25)
Hip adductor tendinopathy	4 (1)	3	3	0	0 (0)
Peroneal tendinopathy	4 (1)	1	2	0	0 (0)
Fracture tibia	3 (1)	0	1	1	0 (0)
Fracture 5^th^ metatarsal bone	3 (1)	0	2	0	0 (0)
Osteoarthritis knee	3 (1)	2	0	1	3 (100)
Pes ancerinus tendinopathy	3 (1)	0	2	0	0 (0)
Tensor fascia latae tendinopathy	3 (1)	1	0	1	1 (33)
Spinal injuries	2 (<1)	1	2	0	0 (0)
Fracture navicular	2 (<1)	1	0	1	0 (0)
Fracture calcaneus	2 (<1)	2	1	0	1 (50)
Fracture fibula	2 (<1)	2	0	0	0 (0)
Unknown	4 (1)	2	0	0	0 (0)
Other	21 (8)	8 (0.38)	3 (<0.01)*	8	3 (14)
**Total**	**254**			**63**	**34 (13.4)**

*Legend*: Injuries presented in descending order with the most frequent presented first. p =  p-value. In males, the count of males of the total count are presented and the p-value (p) from the test (exact binomial probability test) that the proportion of males is equal to 50%. * =  statistically significant from 50%. In case the assumptions behind the exact test are violated (count <10), the p-values are not presented. Similarly, the proportion of individuals with an age above 40 and the test for a proportion equal to 50% is presented. In addition, the number of bilateral injuries and the number and percentage of injuries not recovering is presented.

A majority of the injuries occurred in the lower leg (n = 94; 37%), knee (n = 82; 32.3%) and ankle/foot (n = 36; 14.2%), followed by the hip (n = 27; 10.6%). Only a minority of injuries occurred in the upper leg (n = 8; 3.2%) and lower back (n = 3; 1.2%). Finally, four injuries (1.5%) occurred in other anatomical locations than the above.

In Table 2, data on time to recovery in days from injury occurrence to complete recovery is presented. Additionally, the counts and percentages of the injured using medication and receiving conservative treatment are presented. At the last follow-up in February 2013, 36 (3.8%) of the 933 participants originally included in the DANO-RUN study still reported symptoms during activities of daily living as a consequence of their running-related injury. Of these, 20 had been injured for more than 1 year and 7 for more than 1½ years. No difference in time to recovery between males and females (p-value  = 0.98) or age dichotomized based on below and above 40 years (p-value  = 0.69) existed.

**Table 2:** Descriptive data on the time to recovery.

Injury type	Descriptive data on the recovered injuries (n = 220)			
	Time to recovery in days	Used medication	Received conservative treatment	Treated surgically
	Median (min/max)	n (%)	n (%)	n (%)
Medial Tibial Stress Syndrome	72 (16/582)	2 (5)	3 (8)	0 (0)
Patellofemoral Pain	77 (28/399)	4 (15)	2 (8)	1 (4)
Meniscal injury (Medial)	87 (16/362)	8 (35)	5 (22)	7 (30)
Achilles tendinopathy	82 (21/479)	2 (11)	1 (5.5)	0 (0)
Plantar fasciitis	159 (51/308)	0 (0)	2 (17)	1 (8)
Soleus injury	31 (15/115)	2 (17)	1 (8)	0 (0)
Ilio-Tibial Band Syndrome	88 (22/398)	2 (18)	1 (9)	0 (0)
Patella tendinopathy	75 (15/444)	1 (9)	0 (0)	0 (0)
Gastrocnemius injury	40 (9/235)	1 (11)	0 (0)	0 (0)
Gluteus Medius injury	124 (45/317)	1 (12.5)	0 (0)	0 (0)
Hamstring	58 (12/312)	1 (12.5)	1 (12.5)	0 (0)
Ankle inversion	53 (28/128)	2 (28.5)	0 (0)	0 (0)
Compartment (lower leg)	113 (64/572)	1 (17)	1 (17)	1 (17)
Iliopsoas injury	71 (62/368)	1 (20)	0 (0)	0 (0)
Hip trochanteric bursitis	174 (107/235)	0 (0)	1 (25)	0 (0)
Hip adductor tendinopathy	101 (14/228)	2 (50)	3 (75)	0 (0)
Peroneal tendinopathy	86 (20/161)	3 (75)	0 (0)	0 (0)
Fracture tibia	145 (19/369)	0 (0)	0 (0)	0 (0)
Fracture 5^th^ metatarsal bone	65 (50/268)	0 (0)	0 (0)	0 (0)
Osteoarthritis knee	n/a	0 (0)	0 (0)	0 (0)
Pes ancerinus tendinopathy	32 (24/32)	1 (33)	0 (0)	0 (0)
Tensor fascia latae tendinopathy	26 (22/31)	1 (33)	0 (0)	0 (0)
Spinal injuries	99	2 (100)	0 (0)	0 (0)
Fracture navicular	67	0 (0)	1 (50)	0 (0)
Fracture calcaneus	66	0 (0)	1 (50)	0 (0)
Fracture fibula	76	1 (50)	1 (50)	0 (0)
Unknown	104 (16/173)	1 (25)	0 (0)	0 (0)
Other	56 (14/214)	7 (33)	3 (14)	2 (10)
**Total**	**71 (9/582)**	**46 (18.2)**	**27 (10.7)**	**12 (4.7)**

*Legend*: time to recovery was measured in days from injury occurrence to complete recovery. In addition, the counts and percentage of the participants using medication, receiving conservative treatment and who were treated surgically are presented. n/a =  data not available. Min  =  minimum. Max  =  maximum. In time to recovery, no data is presented for minimum and maximum values if the total numbers of injuries are below 3.

Among those recovered from their injury, ten runners missed days from work because of their injury (range 1 day to 14 days). The diagnoses which caused missed days at work were mainly medial meniscal injury (n = 3) and stress fractures (n = 3), but also ankle inversion injury (n = 1), anterior compartment (n = 1), Achilles tendinopathy (n = 1) and gastrocnemius injury (n = 1). Among 203 participants reporting information about their motivation to start running after full recovery, 22 participants were less motivated or not at all motivated to start running again. These participants had an almost significantly longer time to recovery (log transformed) of 0.36 days (95% CI: −0.02; 0.74), *p* = 0.06 than those motivated to start running after having had an injury.

## Discussion

Medial tibial stress syndrome was the most common injury among novice runners followed by patellofemoral pain and injury in the medial meniscus. Depending on injury type, injured novice runners should expect a median time to recovery of approximately 10 weeks. Of the 933 healthy novice runners originally included in the DANO-RUN study, 4% remained injured (mainly: meniscal injury n = 6; patellofemoral pain n = 3; plantar fasciitis n = 3) at the end of follow-up and had to permanently stop running because of their injury.

To our knowledge, the present study is the first to provide insight into the time to recovery for all types of running-related injuries. Based on the results presented in Table 2, considerable variation in time to recovery existed across the different types of injuries. Soleus injuries and gastrocnemius injuries had a median time to recovery of 30–40 days, while the recovery was 159 days for plantar fasciitis and 174 days for trochanteric bursitis. Even though the inter-quartile ranges were substantial within diagnosis, clinicians may use the results on time to recovery as prognostic indicator when dealing with injured novice runners. Importantly, other factors than diagnosis should also be included as prognostic indicators like differences in biology, anatomy and training prior to injury. One study has investigated time to recovery in a prospective setup. Moen et al [11] investigated time to recovery in fifteen male subjects from the Dutch army who had medial tibial stress syndrome. The mean time to recovery was 58 days (Standard deviation  = 27 days). This is slightly lower than the time to recovery (median  = 72 days) among the novice runners sustaining medial tibial stress syndrome in the present study. The reason for this discrepancy remains unknown, but it may be related to differences in the two populations or differences in the definition of the time to recovery.

The 254 injured runners described in the present paper were a sub-sample of the 933 volunteers included in the DANO-RUN study [5]. Of the 933 runners taking up running, the present study revealed a proportion of runners remaining injured at the end of follow-up of 4%. These persons had to stop running permanently because of their injury and many of them had symptoms during activities of daily living. This finding underlines the importance of preventing running-related injuries in order to enable individuals to maintain an active lifestyle. This is supported by the findings by Koplan et al. [12]: they followed a cohort of runners over a 10-year period and found injury to be the major reason among males and the third most common reason among females for permanently stop running.

A major strength in the present study is the prospective approach used to gather information about the runners and their injuries. Previously, retrospective studies have been conducted[13]–[15]. Wen suggested, however, that the information provided by participants included in retrospective studies may be affected by recall bias [16]. Such bias is not present in a prospective design.

An additional strength was the use of an early clinical examination to diagnose each injury. In many studies, participants report their injury by mail or telephone without attending an examination[3], [17], [18]. By doing so, the possibility to identify the diagnoses sustained by each injured participant becomes limited. Since the clinical examination was made as soon as possible after injury occurrence, we believe the date of injury occurrence was determined as precisely as possible. In addition, a prospective approach was used to contact each of the injured participants every second or third week during their rehabilitation. This limits the recall bias problems, which may occur in retrospective studies.

At least three major limitations in the present paper must be considered: the purpose of the study, the possibility for information bias based on a clinical examination to diagnose a running-related injury and the injury definition.

The purpose of the present paper was different than the original purpose of the DANO-RUN study. The study originally aimed at investigating if the time to injury occurrence varies among runners with different foot postures and progressions in training distance. Additionally, the role of demographic characteristics and behaviour on injury development has been investigated based on the same dataset. Results from these analyses have been presented elsewhere [6], [7], [19] and more papers are submitted for publication. Based on this, the results presented in the present paper should be considered as a sub-analysis without focus on injury aetiology. Based on this, the results from the present paper must be interpreted with caution. Still, we believe the results may be of interest for clinicians and researchers working with novice runners, injury epidemiology, time to recovery and the consequences of running-related injuries.

Another weakness may be the approach used to diagnose injuries. In the present study, most injuries were diagnosed based on the patient history and by performing a physical examination without the use of diagnostic imaging. Previously, Khan et al. [20] stated that listening to the patient and performing a thorough physical examination remains the mainstays of clinical diagnosis and treatment. A standardized examination procedure was used in each of the following anatomical locations: foot/ankle, lower leg, knee, thigh, hip and back. Furthermore, guidelines for diagnostic criteria were used to classify the injuries into specific diagnoses/types of injuries. In the guideline, the decision to diagnose an injury was partly based on a list of positive and negative findings, which was used to make a diagnosis (Material S1). Unfortunately, it was not always possible to follow these positive and negative findings strictly to make a diagnosis because the clinical decision making is complex and challenging. In addition, the diagnoses were not confirmed by diagnostic imaging in most cases [21]. As an example, the proportion of stress fractures in tibia was low in the current study compared to the findings by Taunton et al [2]. Since radiological imaging is recommended for diagnostics of stress fractures in the lower limb [22], some individuals with medial tibial stress syndrome may have been diagnosed with stress fracture in the tibia if the clinical examination had been assisted by diagnostic imaging.

The injuries occurring in the present study was defined as any musculoskeletal complaint of the lower extremity or back caused by running. It can be argued, that some of the injuries included were not directly caused by running. For instance, knee osteoarthritis and injury in the medical meniscus may be caused by other factors and running leads the injuries/conditions to become symptomatic. Since all participants in the study reported themselves as healthy at baseline, such injuries can only be excluded at baseline by performing a diagnostic imaging of all subjects prior to inclusion. This procedure was not used in the present study because of financial reasons.

In addition to the weaknesses presented, it must be stressed that the participants included in the present study had to run in a neutral running shoe. It, therefore, remains unclear if the types of running-related injury are different among novice runners using other types of conventional shoes or minimalist shoes.

## Conclusions

In the present study, medial tibial stress syndrome was the most common injury diagnosed in 38 of 254 injured beginners (15%). In a review by Lopes et al [1] including studies on all types of runners, the medial tibial stress syndrome was also the most common injury with an injury incidence of 13.6% to 20.0%. Based on this, it may be relevant to specifically focus on prevention and treatment of medial tibial stress syndrome since this injury is the most common. Despite the median time to recovery of 72 days, the consequences of this condition were, fortunately, minor: Nearly all participants (97.4%) with medial tibial stress syndrome recovered, none were treated surgically and none missed days from work because of their injury. This may indicate that the consequences of medial tibial stress syndrome are minor compared to other types of injuries. In contrast, medial meniscus injuries lead to 30% receiving surgical treatment, 22% seeking conservative treatment and 26% remained injured at the end of follow-up. Since meniscus injury also was the third most common diagnose (9% of all injuries) more attention towards prevention and treatment of this particular injury may be of significant relevance.

## Supporting Information

Material S1 **Non-validated guidelines to classify the injuries into specific diagnoses/types of injuries.**
(DOCX)

## References

[1] Lopes AD, Hespanhol Junior LC, Yeung SS, Costa LO (2012) What are the main running-related musculoskeletal injuries?: A systematic review. Sports Med 42: 891–905. 22827721 10.1007/BF03262301 PMC4269925

[2] Taunton JE, Ryan MB, Clement DB, McKenzie DC, Lloyd-Smith DR, et al (2002) A retrospective case-control analysis of 2002 running injuries. Br J Sports Med 36: 95–101. 11916889 10.1136/bjsm.36.2.95 PMC1724490

[3] Buist I, Bredeweg SW, van Mechelen W, Lemmink KA, Pepping GJ, et al (2008) No effect of a graded training program on the number of running-related injuries in novice runners: A randomized controlled trial. Am J Sports Med 36: 33–39. 17940147 10.1177/0363546507307505

[4] Taunton JE, Ryan MB, Clement DB, McKenzie DC, Lloyd-Smith DR, et al (2003) A prospective study of running injuries: The vancouver sun run “in training” clinics. Br J Sports Med 37: 239–244. 12782549 10.1136/bjsm.37.3.239 PMC1724633

[5] Nielsen RO, Ramskov D, Sørensen H, Lind M, Rasmussen S, et al (2011) Protocol for the dano-run study: A 1-year observational follow up study on running related injuries in 1000 novice runners. Br J Sports Med 45: 365.

[6] Nielsen RO, Buist I, Parner ET, Nohr EA, Sorensen H, et al (2014) Foot pronation is not associated with increased injury risk in novice runners wearing a neutral shoe: A 1-year prospective cohort study. Br J Sports Med 48: 440–447. 23766439 10.1136/bjsports-2013-092202

[7] Nielsen RO, Buist I, Parner ET, Nohr EA, Sørensen H, et al (2013) Predictors of running-related injuries among 930 novice runners: A 1-year prospective follow-up study. Orthopaedic Journal of Sports Medicine 1: 1–7. 10.1177/2325967113487316 PMC4555503 26535228

[8] Buist I, Bredeweg SW, Bessem B, van Mechelen W, Lemmink KA, et al (2010) Incidence and risk factors of running-related injuries during preparation for a 4-mile recreational running event. Br J Sports Med 44: 598–604. 18487252 10.1136/bjsm.2007.044677

[9] Buist I, Bredeweg SW, Lemmink KA, van Mechelen W, Diercks RL (2010) Predictors of running-related injuries in novice runners enrolled in a systematic training program: A prospective cohort study. Am J Sports Med 38: 273–280. 19966104 10.1177/0363546509347985

[10] McKean KA, Manson NA, Stanish WD (2006) Musculoskeletal injury in the masters runners. Clin J Sport Med 16: 149–154. 16603885 10.1097/00042752-200603000-00011

[11] Moen MH, Bongers T, Bakker EW, Zimmermann WO, Weir A, et al (2012) Risk factors and prognostic indicators for medial tibial stress syndrome. Scand J Med Sci Sports 22: 34–39. 20561280 10.1111/j.1600-0838.2010.01144.x

[12] Koplan JP, Rothenberg RB, Jones EL (1995) The natural history of exercise: A 10-yr follow-up of a cohort of runners. Med Sci Sports Exerc 27: 1180–1184. 7476063

[13] Rasmussen CH, Nielsen RO, Juul MS, Rasmussen S (2013) Weekly running volume and risk of running-related injuries among marathon runners. Int J Sports Phys Ther 8: 111–120. 23593549 PMC3625790

[14] Nielsen RO, Buist I, Sorensen H, Lind M, Rasmussen S (2012) Training errors and running related injuries: A systematic review. Int J Sports Phys Ther 7: 58–75. 22389869 PMC3290924

[15] Knobloch K, Yoon U, Vogt PM (2008) Acute and overuse injuries correlated to hours of training in master running athletes. Foot Ankle Int 29: 671–676. 18785416 10.3113/FAI.2008.0671

[16] Wen DY (2007) Risk factors for overuse injuries in runners. Curr Sports Med Rep 6: 307–313. 17883966

[17] Van Middelkoop M, Kolkman J, Van Ochten J, Bierma-Zeinstra SM, Koes BW (2008) Risk factors for lower extremity injuries among male marathon runners. Scand J Med Sci Sports 18: 691–697. 18266787 10.1111/j.1600-0838.2007.00768.x

[18] Van Middelkoop M, Kolkman J, Van Ochten J, Bierma-Zeinstra SM, Koes BW (2007) Course and predicting factors of lower-extremity injuries after running a marathon. Clin J Sport Med 17: 25–30. 17304002 10.1097/JSM.0b013e3180305e4d

[19] Bertelsen ML, Jensen JF, Nielsen MH, Nielsen RO, Rasmussen S (2013) Footstrike patterns among novice runners wearing a conventional, neutral running shoe. Gait Posture 38: 354–356. 23280125 10.1016/j.gaitpost.2012.11.022

[20] Khan KM, Tress BW, Hare WS, Wark JD (1998) Treat the patient, not the x-ray: Advances in diagnostic imaging do not replace the need for clinical interpretation. Clin J Sport Med 8: 1–4. 9448948

[21] Teh J, Suppiah R, Sharp R, Newton J (2011) Imaging in the assessment and management of overuse injuries in the foot and ankle. Semin Musculoskelet Radiol 15: 101–114. 21332023 10.1055/s-0031-1271962

[22] Schneiders AG, Sullivan SJ, Hendrick PA, Hones BD, McMaster AR, et al (2012) The ability of clinical tests to diagnose stress fractures: A systematic review and meta-analysis. J Orthop Sports Phys Ther 42: 760–771. 22813530 10.2519/jospt.2012.4000

