# Supplementary material for: A Prospective Study on Time to Recovery in 254 Injured Novice Runners
Source: PLoS One. 2014 Jun 12;9(6):e99877. doi: 10.1371/journal.pone.0099877 (PMC4055729; doi:10.1371/journal.pone.0099877)
Supplement: Material S1 — Non-validated guidelines to classify the injuries into specific diagnoses/types of injuries. (DOCX) [file pone.0099877.s001.docx]

**Supplementary material:**

**Non-validated procedures for injury classification**

**Aim of appendix**

The aim of this appendix is to describe the procedures used in the clinical examinations of the injured novice runners in the DANO-RUN study.

**Injury definition**

A running-related injury was defined as any musculoskeletal complaint of the lower extremity or back caused by running, which restricted the amount of running (distance, duration, pace, or frequency) for at least 1 week(1).

**Musculoskeletal examination in selected anatomical sites**

The musculoskeletal examination of the injured subjects described in Gross (2) and Brukner (3) will be used for diagnostic purposes. The examination include observation, a subjective examination (patient anamnesis), and an objective examination. The objective examination include palpation of symptomatic and asymptomatic structures, active movement testing, passive movement testing, resistive testing, neurological examination, and special tests (flexibility, stability, balance, alignment). The physiotherapist and/or doctor may to use additional tests if necessary.

Using the findings from Taunton (4) it is expected that the most common site of injury occurrence is the knee (42 % of all injuries), followed by the foot/ankle (17 %), by the lower leg (13 %), by the hip/pelvis (11 %), by the Achilles/calf (6 %), by the upper leg (5 %), and finally by the lower back (3 %). Other sites did only represent a minority of the total injuries (3 %). Therefore, the procedures used on these anatomical sites are presented below.

*Knee, calf, and upper leg*

Jump on one leg. Deep squat on two legs. “But kickers”. Palpation of medial and lateral borders of patella, apex patella, patella tendon, tibia tuberosity, lateral and medial jointline, medial collateral ligament (MCL), lateral collateral ligament (LCL), insertio pes ancerinus, distal part of tendo tractus iliotibialis including gerdy tuberosity. Tests for Medial Collateral Ligament- (MCL), Lateral Collateral Ligament- (LCL), and Anterior Cruciate Ligament (ACL) laxity. McMurray and Appleys test for medial/lateral meniscal damage/tear. Insall tests for patello-femoral pain syndrome (compression of patella, isometric and dynamic resistance tests for m. quadriceps). Resistance tests m. quadriceps, adductors, and biceps femoris / semimembranosus / semitendinosis / gracilis. Percussion patella and tibia tuberosity.

*Foot/ankle*

Jump on one leg. Deep squat on two legs. Palpation of jointline of art. talocruralis, ligamentum deltoideum, ligamentum talo-fibulare anterior, fascia plantaris including origo, tendons from m. tibialis anterior, tibialis posterior, mm. peroneii, and achilles. Resistance tests m. tibialis anterior, tibialis posterior, mm. peroneii, and achilles.

*Lower leg including Achilles tendon*

Jump on one leg. Deep squat on two legs. Palpation of medial border of tibia 2 to 20 cm above medial malleoli, tibia, m. tibialis anterior, soft tissue of soleus, gastrocnemius, and Achilles tendon. Percussion of tibia and lateral malleoli. Resistance tests of m. tibialis posterior, tibialis anterior, and peroneii. Thomson test.

*Hip/pelvis*

Jump on one leg. Deep squat on two legs. Palpation of greater trochanter, spina iliaca anterior superior, iliac crest, spina iliaca posterior superior, Sacro-Iliac joint. Passive movement internal and external rotation. Compression art. coxae. Resistance tests m. iliopsoas, tensor fascia latae (TFL), adductures, gluteus medius, gluteus maximus. Neurological tests: Straight leg raise (SLR) and SLUMP.

*Other*

No standardised procedure is described. The therapist and/or doctor choose themselves which tests are relevant.

**Injury classification**

According to Taunton (4) the following 20 diagnosis are the most common running injuries. The injuries are presented with the most common diagnosis first. The expected result from the musculoskeletal examination is defined as (P) for positive or (N) for negativ response.

1. *Patello-Femoral Pain Syndrom (Location: knee, frequency: 16.5 %)*

Classification based on findings from Fredericson (6). Jump on one leg (P). Deep squat on two legs (P). “But kickers” (N). Palpation of medial and lateral borders of patella (P), apex patella (N), patella tendon (N), tibia tuberosity (N), lateral and medial jointline (N), medial collateral ligament (N), lateral collateral ligament (N), insertio pes ancerinus (N), distal part of tendo tractus iliotibialis including gerdy tuberosity (N). Tests for Medial Collateral Ligament- (MCL), Lateral Collateral Ligament- (LCL), and Anterior Cruciate Ligament (ACL) laxity (N). McMurray and Appleys test for medial/lateral meniscal damage/tear (N). Insall tests for patello-femoral pain syndrome (compression of patella, isometric and dynamic resistance tests for m. quadriceps) all (P). Resistance tests m. quadriceps (P), adductors (N), and biceps femoris / semimembranosus / semitendinosis / gracilis (N). Percussion patella and tibia tuberosity (N).

1. *Iliotibial band friction syndrome (Location: Knee, frequency: 8.4 %)*

Classification based on findings from Ellis and Khaund (7;8). Jump on one leg (P). Deep squat on two legs (N). “But kickers” (N). Palpation of medial and lateral borders of patella (N), apex patella (N), patella tendon (N), tibia tuberosity (N), lateral and medial jointline (N), medial collateral ligament (N), lateral collateral ligament (N), insertio pes ancerinus (N), distal part of tendo tractus iliotibialis including gerdy tuberosity (P). Tests for Medial Collateral Ligament- (MCL), Lateral Collateral Ligament- (LCL), and Anterior Cruciate Ligament (ACL) laxity (N). McMurray and Appleys test for medial/lateral meniscal damage/tear (N). Insall tests for patello-femoral pain syndrome (compression of patella, isometric and dynamic resistance tests for m. quadriceps) all (N). Resistance tests m. quadriceps, adductors, and biceps femoris / semimembranosus / semitendinosis / gracilis (N). Percussion patella and tibia tuberosity (N).

1. *Plantar fasciitis (Location: Foot, frequency: 7.9 %)*

Classification based on findings from Neufelt (9). Jump on one leg (P). Deep squat on two legs (N). Palpation of jointline of art. talocruralis (N), ligamentum deltoideum (N), ligamentum talo-fibulare anterior (N), fascia plantaris including origo (P), tendons from m. tibialis anterior (N), tibialis posterior (N), mm. peroneii (N), and Achilles (N). Resistance tests m. tibialis anterior (N), tibialis posterior (N), mm. peroneii (N), and Achilles (N).

1. *Meniscal injuries (Location: Knee, frequency: 5.0 %)*

Classification based on recommendations from Brukner (3). Jump on one leg (P). Deep squat on two legs (P). “But kickers” (P). Palpation of medial and lateral borders of patella (N), apex patella (N), patella tendon (N), tibia tuberosity (N), lateral and medial jointline (P), medial collateral ligament (N), lateral collateral ligament (N), insertio pes ancerinus (N), distal part of tendo tractus iliotibialis including gerdy tuberosity (N). Tests for Medial Collateral Ligament- (MCL), Lateral Collateral Ligament- (LCL), and Anterior Cruciate Ligament (ACL) laxity (N). McMurray and Appleys test for medial/lateral meniscal damage/tear (P). Insall tests for patello-femoral pain syndrome (compression of patella, isometric and dynamic resistance tests for m. quadriceps) all (N). Resistance tests m. quadriceps, adductors, and biceps femoris / semimembranosus / semitendinosis / gracilis (N). Percussion patella and tibia tuberosity (N).

1. *Tibial Stress Syndrome (Location: Lower leg, frequency: 5.0 %)*

Classification based on findings from Moen (10). Jump on one leg (P). Deep squat on two legs (N). Palpation of medial border of tibia 2 to 20 cm above medial malleoli (P), tibia (N), m. tibialis anterior (N), soft tissue of soleus (N), gastrocnemius (N), and tendon of achilles (N). Percussion of tibia and lateral malleoli (N). Resistance tests of m. tibialis posterior (P), tibialis anterior (N), and peroneii (N). Thomson test (N).

1. *Patellar tendinitis (Location: Knee, frequency: 4.8 %)*

Classification based on findings from Peers (11). Jump on one leg (P). Deep squat on two legs (P). “But kickers” (P). Palpation of medial and lateral borders of patella (N), apex patella (P), patella tendon (P), tibia tuberosity (P), lateral and medial jointline (N), medial collateral ligament (N), lateral collateral ligament (N), insertio pes ancerinus (N), distal part of tendo tractus iliotibialis including gerdy tuberosity (N). Tests for Medial Collateral Ligament- (MCL), Lateral Collateral Ligament- (LCL), and Anterior Cruciate Ligament (ACL) laxity (N). McMurray and Appleys test for medial/lateral meniscal damage/tear (N). Insall tests for patello-femoral pain syndrome (compression of patella, isometric and dynamic resistance tests for m. quadriceps) all (N). Resistance tests m. quadriceps (P), adductors (N), and biceps femoris / semimembranosus / semitendinosis / gracilis (N). Percussion patella and tibia tuberosity (N).

1. *Achilles tendinitis (Location: Lower leg, frequency: 4.8 %)*

Classification based on findings from Longo (12). Jump on one leg (P). Deep squat on two legs (P). Palpation of medial border of tibia 2 to 20 cm above medial malleoli (N), tibia (N), m. tibialis anterior (N), soft tissue of soleus (N), gastrocnemius (N), and tendon of Achilles (P). Percussion of tibia and lateral malleoli (N). Resistance tests of m. tibialis posterior (N), tibialis anterior (N), and peroneii (N). Thomson test (N).

1. *Gluteus medius injuries (Location: Hip / pelvis, frequency: 3.5 %)*

Classification based on recommendations from Brukner (3). Jump on one leg (P). Deep squat on two legs (P). Palpation of greater trochanter (P), spina iliaca anterior superior (N), iliac crest (N), spina iliaca posterior superior (N), Sacro-Iliac joint (N). Passive movement internal and external rotation (N). Compression art. coxae (N). Resistance tests m. iliopsoas (N), tensor fascia latae (N), adductures (N), gluteus medius (P), gluteus maximus (N). Neurological tests: Straight leg raise (N) and SLUMP (N).

1. *Stress fracture tibia (Location: Lower leg, frequency: 3.3 %)*

Classification based on recommendations from Brukner (3). Jump on one leg (P). Deep squat on two legs (P). Palpation of medial border of tibia 2 to 20 cm above medial malleoli (P), tibia (P), m. tibialis anterior (N), soft tissue of soleus (N), gastrocnemius (N), and tendon of Achilles (N). Percussion of tibia (P) and lateral malleoli (N). Resistance tests of m. tibialis posterior (N), tibialis anterior (N), and peroneii (N). Thomson test (N).

1. *Spinal injuries (Location: lower back, frequency: 2.3 %)*

A medical doctor will diagnose these patients. No description made.

1. *Hamstring injuries (Location: calf, frequency: 2.3 %)*

Classification based on findings from Heiderscheit (13). Jump on one leg (P). Deep squat on two legs (N). “But kickers” (P). Palpation of medial and lateral borders of patella (N), apex patella (N), patella tendon (N), tibia tuberosity (N), lateral and medial jointline (N), medial collateral ligament (N), lateral collateral ligament (N), insertio pes ancerinus (N), distal part of tendo tractus iliotibialis including gerdy tuberosity (N). Tests for Medial Collateral Ligament- (MCL), Lateral Collateral Ligament- (LCL), and Anterior Cruciate Ligament (ACL) laxity (N). McMurray and Appleys test for medial/lateral meniscal damage/tear (N). Insall tests for patello-femoral pain syndrome (compression of patella, isometric and dynamic resistance tests for m. quadriceps) all (N). Resistance tests m. quadriceps (N), adductors (N), and biceps femoris / semimembranosus / semitendinosis / gracilis (P). Percussion patella and tibia tuberosity (N).

1. *Metatarsalgia (Location: foot, frequency: 1.7 %)*

Classification based on recommendations from Brukner (3). Jump on one leg (P). Deep squat on two legs (N). Palpation of jointline of art. talocruralis (N), ligamentum deltoideum (N), ligamentum talo-fibulare anterior (N), fascia plantaris including origo (N), tendons from m. tibialis anterior (N), tibialis posterior (N), mm. peroneii (N), and Achilles (N). Resistance tests m. tibialis anterior (N), tibialis posterior (N), mm. peroneii (N), and Achilles (N). Compression forefoot (P).

1. *Anterior Compartment Syndrome (Location: Lower leg, frequency: 1.4 %)*

Classification based on recommendations from Brukner (3). Jump on one leg (N). Deep squat on two legs (N). Palpation of medial border of tibia 2 to 20 cm above medial malleoli (N), tibia (N), m. tibialis anterior (N), soft tissue of soleus (N), gastrocnemius (N), and tendon of Achilles (N). Percussion of tibia and lateral malleoli (N). Resistance tests of m. tibialis posterior (N), tibialis anterior (N), and peroneii (N). Thomson test (N).

1. *Gastrocnemius injuries (location: Lower leg, frequency: 1.4 %)*

Classification based on recommendations from Brukner (3). Jump on one leg (P). Deep squat on two legs (N). Palpation of medial border of tibia 2 to 20 cm above medial malleoli (N), tibia (N), m. tibialis anterior (N), soft tissue of soleus (N), gastrocnemius (P), and tendon of Achilles (N). Percussion of tibia and lateral malleoli (N). Resistance tests of m. tibialis posterior (N), tibialis anterior (N), and peroneii (N). Thomson test (N).

1. *Greater trochanteric bursitis (Location: Hip / pelvis, frequency: 1.2 %)*

Classification based on findings from Williams (14). Jump on one leg (P). Deep squat on two legs (N). Palpation of greater trochanter (P), spina iliaca anterior superior (N), iliac crest (N), spina iliaca posterior superior (N), Sacro-Iliac joint (N). Passive movement internal and external rotation (N). Compression art. coxae (N). Resistance tests m. iliopsoas (N), tensor fascia latae (N), adductures (N), gluteus medius (P), gluteus maximus (N). Neurological tests: Straight leg raise (N) and SLUMP (N).

1. *Adductor injuries (Location: Upper leg, frequency: 1.1 %)*

Classification based on recommendations from Brukner (3). Jump on one leg (P). Deep squat on two legs (N). “But kickers” (N). Palpation of medial and lateral borders of patella (N), apex patella (N), patella tendon (N), tibia tuberosity (N), lateral and medial jointline (N), medial collateral ligament (N), lateral collateral ligament (N), insertio pes ancerinus (N), distal part of tendo tractus iliotibialis including gerdy tuberosity (N). Tests for Medial Collateral Ligament- (MCL), Lateral Collateral Ligament- (LCL), and Anterior Cruciate Ligament (ACL) laxity (N). McMurray and Appleys test for medial/lateral meniscal damage/tear (N). Insall tests for patello-femoral pain syndrome (compression of patella, isometric and dynamic resistance tests for m. quadriceps) all (N). Resistance tests m. quadriceps (N), adductors (P), and biceps femoris / semimembranosus / semitendinosis / gracilis (N). Percussion patella and tibia tuberosity (N).

1. *Osteoarthritis knee (Location: Knee, frequency: 1.1 %)*

Classification based on recommendations from Brukner (3). Jump on one leg (P). Deep squat on two legs (P). “But kickers” (N). Palpation of medial and lateral borders of patella (N), apex patella (N), patella tendon (N), tibia tuberosity (N), lateral and medial jointline (P), medial collateral ligament (N), lateral collateral ligament (N), insertio pes ancerinus (N), distal part of tendo tractus iliotibialis including gerdy tuberosity (N). Tests for Medial Collateral Ligament- (MCL), Lateral Collateral Ligament- (LCL), and Anterior Cruciate Ligament (ACL) laxity (N). McMurray and Appleys test for medial/lateral meniscal damage/tear (N). Insall tests for patello-femoral pain syndrome (compression of patella, isometric and dynamic resistance tests for m. quadriceps) all (N). Resistance tests m. quadriceps (N), adductors (N), and biceps femoris / semimembranosus / semitendinosis / gracilis (N). Percussion patella and tibia tuberosity (N).

1. *Sacroiliac injuries (Location: Knee, frequency: 1.1 %)*

A surgeon will diagnose these patients. No description made.

1. *Stress fracture femur (Location: Upper leg, frequency: 1.0 %)*

Classification based on recommendations from Brukner (3). Jump on one leg (P). Deep squat on two legs (N). “But kickers” (N). Palpation of medial and lateral borders of patella (N), apex patella (N), patella tendon (N), tibia tuberosity (N), lateral and medial jointline (N), medial collateral ligament (N), lateral collateral ligament (N), insertio pes ancerinus (N), distal part of tendo tractus iliotibialis including gerdy tuberosity (N). Tests for Medial Collateral Ligament- (MCL), Lateral Collateral Ligament- (LCL), and Anterior Cruciate Ligament (ACL) laxity (N). McMurray and Appleys test for medial/lateral meniscal damage/tear (N). Insall tests for patello-femoral pain syndrome (compression of patella, isometric and dynamic resistance tests for m. quadriceps) all (N). Resistance tests m. quadriceps (P), adductors (P), and biceps femoris / semimembranosus / semitendinosis / gracilis (N). Percussion patella and tibia tuberosity (N).

1. *Ankle inversion injuries (Location: Ankle, frequency: 0.9 %)*

Classification based on recommendations from Brukner (3). Jump on one leg (P). Deep squat on two legs (N). Palpation of jointline of art. talocruralis (P), ligamentum deltoideum (P), ligamentum talo-fibulare anterior (P), fascia plantaris including origo (N), tendons from m. tibialis anterior (N), tibialis posterior (N), mm. peroneii (N), and Achilles (N). Resistance tests m. tibialis anterior (N), tibialis posterior (N), mm. peroneii (N), and Achilles (N).

Reference List

(1) Buist I, Bredeweg SW, van MW, Lemmink KA, Pepping GJ, Diercks RL. No effect of a graded training program on the number of running-related injuries in novice runners: a randomized controlled trial. Am J Sports Med 2008 Jan;36(1):33-9.

(2) Gross J, Fetto J, Rosen E. Musculoskeletal examination. 2 nd ed. Blackwell Science; 2002.

(3) Brukner P, Khan K. Clinical Sports Medicine. 3 rd. ed. McGraw-Hill Australia Pty Ltd; 2006.

(4) Taunton JE, Ryan MB, Clement DB, McKenzie DC, Lloyd-Smith DR, Zumbo BD. A retrospective case-control analysis of 2002 running injuries. Br J Sports Med 2002 Apr;36(2):95-101.

(5) Khan KM, Tress BW, Hare WS, Wark JD. Treat the patient, not the x-ray: advances in diagnostic imaging do not replace the need for clinical interpretation. Clin J Sport Med 1998 Jan;8(1):1-4.

(6) Fredericson M, Yoon K. Physical examination and patellofemoral pain syndrome. Am J Phys Med Rehabil 2006 Mar;85(3):234-43.

(7) Ellis R, Hing W, Reid D. Iliotibial band friction syndrome--a systematic review. Man Ther 2007 Aug;12(3):200-8.

(8) Khaund R, Flynn SH. Iliotibial band syndrome: a common source of knee pain. Am Fam Physician 2005 Apr 15;71(8):1545-50.

(9) Neufeld SK, Cerrato R. Plantar fasciitis: evaluation and treatment. J Am Acad Orthop Surg 2008 Jun;16(6):338-46.

(10) Moen MH, Tol JL, Weir A, Steunebrink M, De Winter TC. Medial tibial stress syndrome: a critical review. Sports Med 2009;39(7):523-46.

(11) Peers KH, Lysens RJ. Patellar tendinopathy in athletes: current diagnostic and therapeutic recommendations. Sports Med 2005;35(1):71-87.

(12) Longo UG, Ronga M, Maffulli N. Achilles tendinopathy. Sports Med Arthrosc 2009 Jun;17(2):112-26.

(13) Heiderscheit BC, Sherry MA, Silder A, Chumanov ES, Thelen DG. Hamstring strain injuries: recommendations for diagnosis, rehabilitation, and injury prevention. J Orthop Sports Phys Ther 2010 Feb;40(2):67-81.

(14) Williams BS, Cohen SP. Greater trochanteric pain syndrome: a review of anatomy, diagnosis and treatment. Anesth Analg 2009 May;108(5):1662-70.
